# Competitive adsorptive removal of promazine and promethazine from wastewater using olive tree pruning biochar: operational parameters, kinetics, and equilibrium investigations

**DOI:** 10.1007/s11356-023-27688-6

**Published:** 2023-06-16

**Authors:** Marwa El-Azazy, Ahmed S. El-Shafie, Samer Fawzy, David W. Rooney, Ahmed I. Osman

**Affiliations:** 1 Present Address: Department of Chemistry and Earth Sciences, College of Arts and Sciences, Qatar University, 2713 Doha, Qatar; 2 School of Chemistry and Chemical Engineering, Queen’s University Belfast, David Keir Building, Stranmillis Road, Belfast, BT9 5AG Northern Ireland UK; 3 Chemistry Department, Faculty of Science, South Valley University, Qena, 83523 Egypt

**Keywords:** Wastewater treatment, Olive tree pruning biochar, Phenothiazines, Central composite design (CCD), Binary mixture

## Abstract

This research aims to remove two phenothiazines, promazine (PRO) and promethazine (PMT), from their individual and binary mixtures using olive tree pruning biochar (BC-OTPR). The impact of individual and combinatory effects of operational variables was evaluated for the first time using central composite design (CCD). Simultaneous removal of both drugs was maximized utilizing the composite desirability function. At low concentrations, the uptake of PRO and PMT from their individual solutions was achieved with high efficiency of 98.64%, 47.20 mg/g and 95.87%, 38.16 mg/g, respectively. No major differences in the removal capacity were observed for the binary mixtures. Characterization of BC-OTPR confirmed successful adsorption and showed that the OTPR surface was predominantly mesoporous. Equilibrium investigations revealed that the Langmuir isotherm model best describes the sorption of PRO/PMT from their individual solutions with maximum adsorption capacities of 640.7 and 346.95 mg/g, respectively. The sorption of PRO/PMT conforms to the pseudo-second-order kinetic model. Regeneration of the adsorbent surface was successfully done with desorption efficiencies of 94.06% and 98.54% for PRO and PMT, respectively, for six cycles.

**Graphical abstract:**

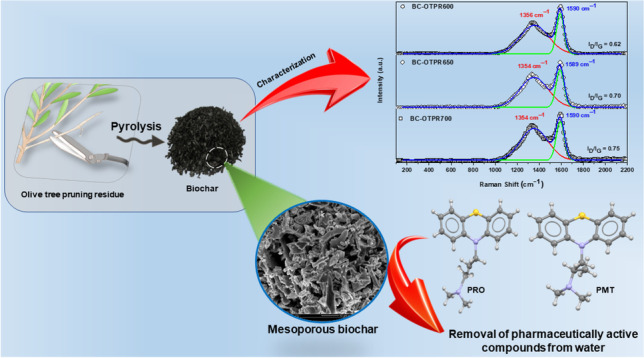

**Supplementary information:**

The online version contains supplementary material available at 10.1007/s11356-023-27688-6.

## Introduction

In the past few decades, contamination of the environment with pharmaceutically active compounds (PhACs) has garnered international attention. Between 2001 and 2019, the global pharmaceutical market's revenue increased from 390 to 1,250 billion dollars. This elevated consumption rate is unavoidably a problem that justifies the presence of PhACs in diverse aquatic environments. Long-term exposure to a substance that exists at negligible concentrations could have deleterious effects on the ecosystem. In general, PhACs do not exist in isolation in the ecosystem; rather, they exist as a multi-component mixture of not only the parent drug compound but also the co-administered drugs and their transformation products. Consequently, PhACs could exhibit synergistic or antagonistic behavior (dos Santos et al. 2022).

Phenothiazines are the largest class of antipsychotic medications. Pharmaceuticals derived from phenothiazine with additional therapeutic effects (e.g., antiparkinsonian, antihistaminic, and antimicrobial alone or in combination with other antibiotics) are widely prescribed (Trautwein and Kümmerer 2012). Promazine (PRO) and promethazine (PMT) are two of the most frequently used phenothiazines. PRO is a psychotropic drug for treating psychiatric and cancer-related conditions, while PMT is an antihistamine with antiemetic properties (Al-Hetlani et al. 2022a; Wilde et al. 2017). The chemical structure and physicochemical properties of PRO and PMT are presented in Table 1 (Domańska et al. 2011; Wilde et al. 2017). When these substances are disposed of directly or indirectly in aquatic environments, they become pervasive contaminants that pose a significant ecological risk.

**Table 1:**
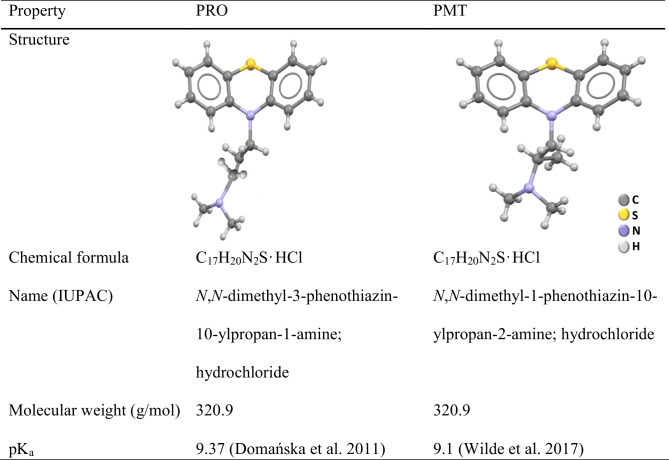
Physicochemical data of PRO and PMT

Literature surveying shows that depollution of pharmaceutical wastewater has been performed employing conventional treatment approaches (e.g., coagulation, sedimentation, sand filtration) as well as advanced processes (e.g., ozonation, adsorption, reverse osmosis) (Cristóvão et al. 2022; El-Azazy et al. 2021b; El-Gendy et al. 2020; Khan et al. 2021; Yang et al. 2017). Among these methods, adsorption onto carbonaceous materials has been widely utilized for the treatment of pharmaceutical wastewater due to its many advantages (high capacity due to the high surface area, speed, absence of byproducts, low investment, and simple operation) (Abdel Maksoud et al. 2020; Osman et al. 2022).

With the growing interest in the concept of a more "circular economy," the valorization of biomass feedstocks into products with added value is the subject of an abundance of research. On the one hand, recycling agro-waste materials that would otherwise end up in a landfill or represent an environmental burden is consistent with the principles of a circular economy. Moreover, biomasses are viewed as a renewable resource and therefore fit well into the never-ending competition for green and sustainable substances for environmental bioremediation (Fawzy et al. 2022b). On the other hand, these materials are predominantly lignocellulosic and, as a result, have a high surface area and are rich in functional moieties with the potential for functionalization, making them excellent candidates for scavenging pollutants (Corona et al. 2019; El-Azazy et al. 2021a; Khajvand et al. 2022; Li et al. 2020; Pang et al. 2019; Xue et al. 2023).

The olive industry is a well-established industrial sector, particularly in the Mediterranean region, with Spain being the largest producer of olive oil. FAO (Food and Agriculture Organization) reported that 21 million tons of olives were harvested from 11 million hectares of land (Corral-Bobadilla et al. 2021; El-Shafie et al. 2022; Fawzy et al. 2022a). With such a vast amount of agricultural land, the amount of waste generated is alarming. Previous research indicates that, for every hectare of olive grove, 3 tons of pruning residues (wood, branches, and leaves) are generated, making them the most abundant byproduct, followed by the extracted pomace byproducts (1.73 tons) and olive stones (0.6 tons). As a significant byproduct, olive tree pruning (OTPR) faces a major fate; incineration, which could negatively contribute to air pollution and soil mineralization (González-Arias et al. 2020). With such massive waste, the olive industry is facing serious sustainability challenges. Literature surveying shows that applications entailing the recycling and utilization of OTPR in wastewater remediation are limited (Anastopoulos et al. 2018, 2015; Blázquez et al. 2011; Calero et al. 2013a), with most of the efforts being devoted to removing inorganic pollutants from their single solution, especially Pb (II). Prior chemical treatment with acids or bases was prevalent in the majority of these studies. Variables influencing the performance of OTPR as a biosorbent were, for the most part, investigated using a univariate design in which only one variable was examined at a time, with all the disadvantages that this approach could bring.

To the best of our knowledge, this is the first investigation into using biochar of OTPR (BC-OTPR) produced at varying pyrolysis temperatures (600–700 °C) to remove two drugs: PRO and PMT. Almost all studies focused on the removal of each drug through the adsorptive treatment of its individual solution (Al-Hetlani et al. 2022a; Barasarathi et al. 2022; D'Cruz et al. 2020b, Seki and Yurdakoç 2009). Therefore, the current approach is novel and possibly the first report on the competitive adsorptive decontamination of PRO and PMT from their binary mixture. The performance of the best biochar in removing PRO and PMT will be analyzed using a response surface methodology—central composite design (CCD) approach, taking advantage of all the benefits of such a design (saving time and resources, retaining method greenness, and generating trustworthy data). Based on the findings of the preliminary study, four variables that affect the adsorption effectiveness: (dose of BC-OTPR (AD), drug concentration [Drug], contact time (CT), and pyrolysis temperature (PyTemp)) will be investigated, with two responses being evaluated: %removal (%R) and adsorption capacity (*q*_*e*_) (Lee et al. 2022).

## Experimental

### Materials and reagents

PRO and PMT were procured from Biosynth® Carbosynth Ltd. (Berkshire, UK). The remaining chemicals were Sigma–Aldrich (MO, USA) commodities. The OTPR biochar employed in the current study was obtained from an agricultural waste management company in Egypt. The three biochar samples prepared at three pyrolysis temperatures (600, 650, and 700 °C) were received in the chopped form, further pulverized, and labelled as BC-OTPR600, BC-OTPR650, and BC-OTPR700. Deionized water (DW) from a Millipore-Q water supply (18.2 MΩ.cm) was used throughout this study. Stock solutions (100 ppm) of PRO and PMT and their further dilutions were prepared in DW and were utilized for the subsequent batch experiments. Aqueous solutions of either hydrochloric acid or sodium hydroxide (0.1 M) were used to adjust the pH to the required value.

### Equipment and software

A centrifuge (Thermo Fisher Scientific, USA) was utilized to separate the adsorbent from the adsorbate. Millex syringe filters (non-sterile 0.45 µm, nylon) were applied to filter the adsorbate–adsorbents suspensions. An Agilent diode-array UV–Vis spectrophotometer (USA) with 10 mm matched quartz cuvette cells were operated to determine the absorbance and hence quantify the drug concentration in the filtrates. To explore the possibility of occurrence of chemisorption, Fourier transform-infrared spectroscopy (FTIR, Perkin Elmer, USA) was employed to determine band positions on the surface of BC-OTPR prior to and following the adsorption of PRO and PMT. Scanning electron microscopy (SEM, Thermo Scientific, USA) with an energy-dispersive X-ray spectrometer (EDX) was used to describe the morphological features and elemental content of BC-OTPR. Raman analysis (Thermo Scientific, USA) was employed to inspect the graphitic structure of BC-OTPR. Surface characteristics in terms of pores and surface area were established employing a Micrometrics ASAP2020 accelerated surface area and Porosimetry system. Minitab®19 (Minitab Inc., PA, USA) was the software operated to assemble and scrutinize the CCD.

### Point of zero charge (pH_PZC_) of BC-OTPR

The pH_PZC_ of the three biochars of OTPR was established by employing the pH drift approach (Babić et al. 1999). A series of 0.01 M sodium chloride solutions were made at initial pH values (pH_initial_) of 3.0 – 9.0 ± 0.2. A mass of 0.20 g of the BC-OTPR (obtained at the different pyrolysis temperatures) was mixed with 25 mL aliquots of 0.01 M NaCl. Samples were retained for 48 h, and the shaker speed was kept constant (150 rpm). The pH of these solutions was then gauged and identified as pH_final_. The pH_PZC_ value was established as the joint point on the curve resulting from plotting pH_initial_ versus pH_final_.

### Batch adsorption experiments: Response surface methodology – CCD

#### Removal of PRO and PMT from their individual solutions

In the current study, optimum process variables were attuned using the central composite design (CCD). Four main effects were examined: AD (A), [PRO] or [PMT] (B), CT (C), and PyTemp (D). In this itinerary, the design consisted of 30 basic runs (16 cube points, 8 axial points, 4 center points in cube, and 2 axial central points) conducted over 2 blocks, with α = 1 (Table S1, Supplementary Materials). The preceding design is a 2-level full factorial design. The target was decided to accomplish the utmost percentage removal (%R) and adsorption capacity (*q*_*e*_, mg/g) (Azari et al. 2023). Dependent responses were resolved by applying Eqs. (1) and (2).

1 $$\left(\mathrm{\%R}\right)= \frac{{\mathrm{C}}_{0}-{\mathrm{C}}_{\mathrm{e}}}{{\mathrm{C}}_{0}}\times 100\mathrm{\%}$$

2 $$\left({q}_{e}\right)= \frac{{\mathrm{C}}_{0}-{\mathrm{C}}_{\mathrm{e}}}{\mathrm{W}}\mathrm{ V}$$

In these two equations, C_0_ signifies the initial concentration of the PRO/PMT solution (ppm), C_e_ expresses the concentration of the PRO/PMT solutions at equilibrium, W is the weight of the adsorbent (g), and V is the volume of PRO/PMT solution (L). Experimental values for both responses, together with the predicted values as well as the calculated relative error (RE), are shown (Table S1, Supplementary Materials). The optimization plot tool was used to decide on the optimum levels for each variable that could maximize the removal of every drug from its individual solution. A value of individual desirability function *(d)* close to 1.0000 was used to judge the efficiency of the variables’ mixture in maximizing each of the measured responses (Derringer and Suich 1980).

#### Removal of PRO and PMT from their binary mixture

To establish the optimal experimental circumstances that maximize the uptake of PRO and PMT simultaneously from their binary mixture, the Derringer ‘composite desirability’ function (*D*) tool provided by Minitab® was used, Eq. (3) (Derringer and Suich 1980, Myers et al. 2009).

3 $$D={\left({d}_{1}^{r1} {d}_{2 }^{r2}\ldots {d}_{m}^{rm}\right)}^{\frac{1}{\sum ri }}={\left(\prod \nolimits_{i=1}^{n}{d}_{1}^{ri}\right)}^{\frac{1}{\sum ri}}$$

where *D* is the composite desirability, *d* is the individual desirability, *r* is the significance of each response, and *m* is the number of responses to be optimized. Acquired individual spectra of PRO and PMT disclosed maximum absorbance at wavelengths 300 and 295 nm, respectively, indicating a case of completely overlapped spectra (Fig. S1, Supplementary Materials). The absorbance of drugs in their binary mixture, A_mix_, is given by Eq. (4):

4 $${\mathrm{A}}_{\mathrm{mix}}= {\varepsilon }_{1}b[\mathrm{PRO}]+ {\varepsilon }_{2}b[\mathrm{PMT}]$$

where ${\varepsilon }_{1}$ and ${\varepsilon }_{2}$, [PRO] and [PMT] are the molar absorptivities and concentrations of PRO and PMT solutions, respectively, therefore:

$${\mathrm{A}}_{\mathrm{PRO}-\mathrm{Std}}= {\varepsilon }_{1}b{[\mathrm{PRO}]}_{\mathrm{Std}}{ ;\mathrm{ A}}_{\mathrm{PMT}-\mathrm{Std}}= {\varepsilon }_{1}b{[\mathrm{PMT}]}_{\mathrm{Std}}$$

where [PRO]_Std_ and [PMT]_Std_ are the concentrations of PRO and PMT standard solutions (Std), respectively. The mixture was measured using wavelengths in the range of 285–310 nm. Substitution of the molar absorptivities into Eq. (4) and rearrangement yields Eq. (5), and the straight-line equation was obtained by plotting A_mix_/A_PRO-Std_ *versus* A_PMT-Std_/A_PRO-Std_, Eq. (5).

5 $$\frac{{\mathrm A}_{\mathrm{mix}}}{{\mathrm A}_{\mathrm{PRO}-\mathrm{Std}}}=\frac{\left[\mathrm{PRO}\right]}{{\left[\mathrm{PRO}\right]}_{\mathrm{Std}}}+\frac{\left[\mathrm{PMT}\right]}{{\left[\mathrm{PMT}\right]}_{\mathrm{Std}}}\cdot\frac{{\mathrm A}_{\mathrm{PMT}-\mathrm{Std}}}{{\mathrm A}_{\mathrm{PRO}-\mathrm{Std}}}$$

where A_PRO-Std_ and A_PMT-Std_ are the absorbances of PRO and PMT standard solutions, respectively, the intercept was used to calculate the unknown [PRO], while the slope was used to calculate the unknown [PMT].

### Equilibrium Investigation

#### Individual solute solutions

For equilibrium studies, stock solutions of PRO and PMT (1000 ppm each) were made in DW. Using appropriate dilutions with the same solvent, the samples were prepared in the 10–800 ppm range. The adsorbent, BC-OTPR700 (0.100 ± 0.005 g), was mixed with the drug solution keeping the final volume at 13 mL. The resultant mixture was left for 24 h using a shaking incubator at a speed of 150 rpm. The mixture was then filtered using a syringe filter, and the absorbance of the filtrate was recorded at 300 and 295 nm for PRO and PMT, respectively.

#### Binary solute solutions

A stock solution of PRO and PMT (1000 ppm of both drugs) was prepared in DW for the binary mixtures. The equilibrium isotherm experiments were performed for diluted solutions (10–800 ppm), and the same procedures mentioned under the individual solutions were followed. The absorbances of the prepared samples were noted at multiple wavelengths (285–310 nm, stepping 5 nm). The residual drug concentrations in the mixture were quantified using Eqs. (4) and (5). Sample calculations for a mixture are shown (Table S2, Supplementary Materials).

### Kinetic studies

To reconnoiter the adsorption kinetics, 200 mL of a 500-ppm drug solution was mixed with 1.0 g of BC-OTPR700 on a magnetic stirrer. Over a duration of 90 min, an aliquot of ~ 10 mL was periodically removed. Following each removal, the mixed solution was filtered using a syringe filter, and the filtrate absorbance was noted at 300 and 295 nm for PRO and PMT, respectively.

### Adsorbent regenration

A desorption investigation study was conducted using five different eluents, followed by six sequential adsorption–desorption cycles. Five eluents were evaluated for the desorption of both PRO and PMT from loaded BC-OTPR700, including 0.1 M solutions of hydrochloric acid, sulfuric acid, and sodium carbonate, in addition to 10% ethanol and water.

## Results and discussion

### Multi-linear regression analysis (MLRA) of PRO-PMT binary mixtures

The current study involves the removal of two drugs from their individual and binary solutions. While determining the residual drug concentrations following their adsorption from their individual solutions is the normal rehearsal, approaching this treatment in a binary mixture system was challenging. MLRA was an ideal solution for the resolution of the closely overlapped spectra of the binary mixtures without involving a chemical pretreatment or a graphical procedure for the overlapping spectra (Blanco et al. 1989; Dinç 2003). For either individual or binary solutions, the absorption spectrum for each investigated drug was perceived between 200–800 nm versus a reagent blank (Fig. S1, Supplementary Materials). MLRA of the completely overlapped spectra of PRO-PMT mixtures was approached using Eqs. (4) and (5), and sample calculations are shown (Table S2, Supplementary Materials).

### Exploration of the statistically substantial effects

**Fig. 1:**
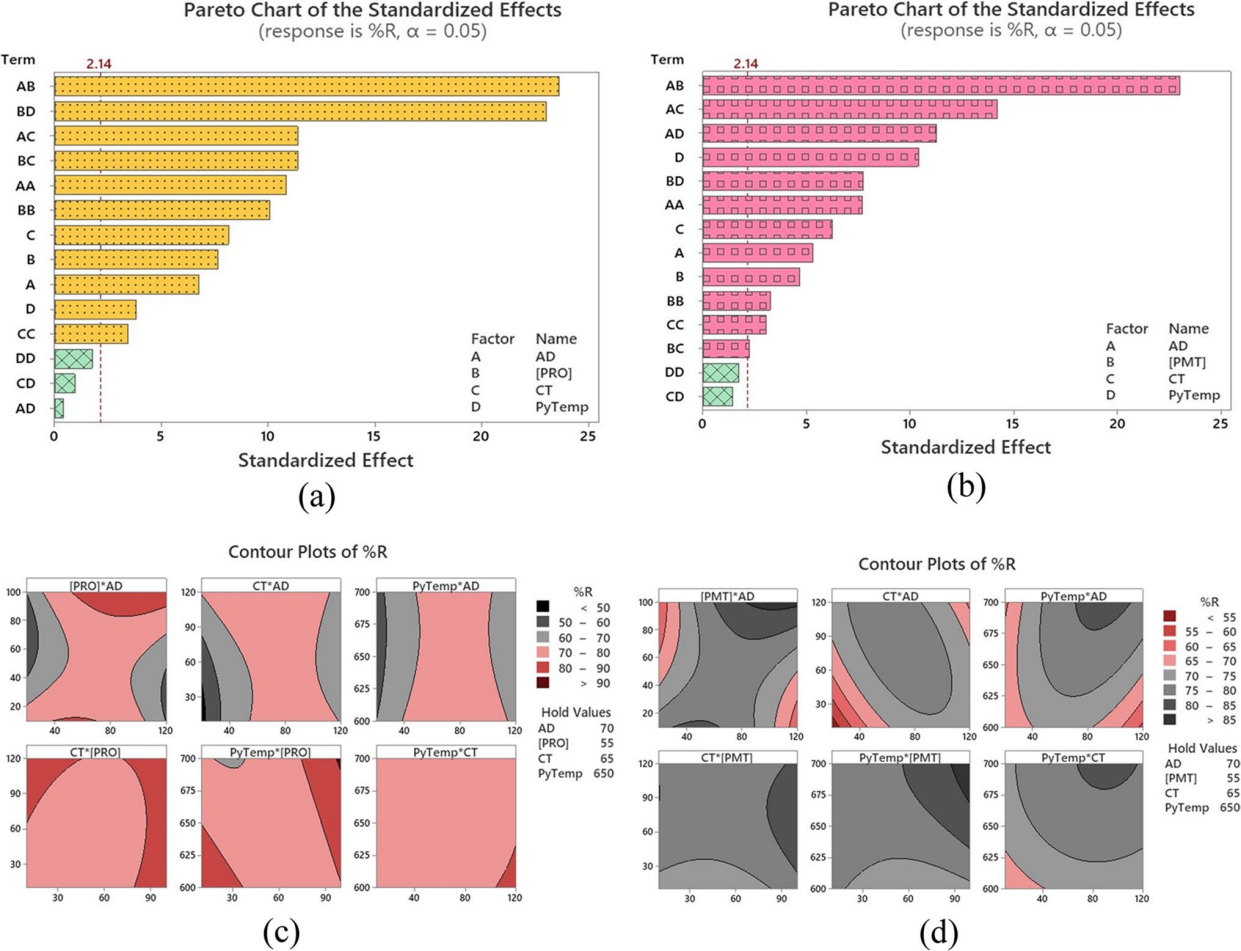
Pareto chart of standardized effects and two-dimensional contour plot. Measured responses are (**a**, **c**) %R_(PRO)_, (**b**, **d**) %R_(PMT)_

To assess the statistical implication and magnitude of the investigated independent variables, Pareto charts of the standardized effects were plotted, as shown in Fig. 1a, b. When the response is %R_(PRO)_ or %R_(PMT)_, the 2-way variable-variable interaction of AD (A)⨯[Drug] (B) was the most statistically substantial variable in either case. In terms of main effects, CT (C) was the most influential in the case of %R_(PRO)_ compared to PyTemp (D) in the case of %R_(PMT)_. The main effects plots (Figures are not shown) show that as the AD (A) increases, the %R of either drug increases. Such behavior continues showing a plateau between 70–100 mg/13 mL. A decrease in %R could be observed following such a plateau. This result may be explained by the increase in vacant active sites and adsorbent-accessible surface area resulting from a rise in the AD. The decrease in %R with the increase in AD could be attributed to a possible agglomeration of the fine adsorbent particles at a high AD and hence a consequent decrease of available surface area and an expansion in diffusional pathlength (Shukla et al. 2002; Tony et al. 2019). Looking at the initial [Drug] impact, it can be observed that at a very low [Drug] of 10 ppm, %R is ~ 80%. This possibly would be ascribed to the low solute concentration compared to the available active sorption sites. At this stage, the first layer of the adsorbate is formed. A decrease in %R with the increase in [Drug] could be seen using up to 50 ppm of the drug, an issue which may be caused by the saturation of the available active sites. Another increase in %R with the increase in [Drug] ˃50 ppm was then observed and might be ascribed to the development of multilayers of the adsorbate on the surface.

### Analysis of variance (ANOVA) and modelling

The findings of Pareto charts were further confirmed using ANOVA. A p-value ˂ 0.05 suggests the statistical implication of an effect at a 95% confidence level. Data analysis has resulted in semi-empirical regression expressions for both responses, Eqs. (6) – (9). These equations describe the correlation between the dependent and the independent variables (main effects, linear and squared interactions). As a result, these equations could be used to easily calculate the overall impact of any variable on the observed response. It is essential to point out that response transformation was achieved utilizing Box-Cox transformation tool (Box and Cox 1964), with a transformation factor λ of 3 in case of %R_(PRO)_, 0.5 in case of *q*_*e* (PRO),_ 2 for %R_(PMT)_, and 0.25 for *q*_*e* (PMT)_.

6 $${\left(\%{\mathrm{R}}^{\uplambda} -1\right)/\left(\lambda \times {\mathrm{g}}^{\left(\lambda -1\right)}\right)}_{\left(\mathrm{PRO}\right)}=541+0.671\, \mathrm{A}-3.850\, \mathrm{B}+0.248\, \mathrm{ C}-1.339\, \mathrm{D}-0.005051\, {\mathrm{A}}^{2}+0.005787\, {\mathrm{B}}^{2}+0.001326\, {\mathrm{C}}^{2}+0.000815\, {\mathrm{D}}^{2}+0.004867\, \mathrm{AB}-0.001920\, \mathrm{AC}-0.000074\, \mathrm{AD}-0.002133\, \mathrm{BC}+0.004747\, \mathrm{BD}-0.000160\, \mathrm{CD}$$

7 $$\sqrt{{{q}_{e}}_{\left(\mathrm{PRO}\right)}}=12.85+0.03797\, \mathrm{A}-0.06041\, \mathrm{B}-0.00733\, \mathrm{C}-0.0295\, \mathrm{ D}+0.000210\, {\mathrm{A}}^{2}-0.000074\, {\mathrm{B}}^{2}+0.000025\, {\mathrm{C}}^{2}+0.000020\, {\mathrm{D}}^{2}-0.000241\, \mathrm{AB}-0.000021\, \mathrm{AC}-0.000112\, \mathrm{AD}-0.000030\, \mathrm{BC}+0.000182\, \mathrm{BD}+0.000012\, \mathrm{CD}$$

8 $$\%{{\mathrm{R}}^{2}}_{\left(\mathrm{PMT}\right)}=-30095-129.3\, \mathrm{A}-211.4\, \mathrm{B}+33.4\, \mathrm{ C}+124.9\, \mathrm{ D}-0.5052\, {\mathrm{A}}^{2}+0.2627\, {\mathrm{B}}^{2}-0.1654\, {\mathrm{C}}^{2}-0.1133\, {\mathrm{D}}^{2}+0.6686\, \mathrm{AB}-0.3380\, \mathrm{AC}+0.2950\, \mathrm{ AD}-0.0588\, \mathrm{ BC}+0.2245\, \mathrm{ BD}+0.0338\, \mathrm{ CD}$$

9 $${{{q}_{e}}^{{~}^{1}\!\left/ \!{~}_{4}\right.}}_{\left(\mathrm{PMT}\right)}= -1.89-0.01786\, \mathrm{A}+0.01181\, \mathrm{B}+0.000049\, \mathrm{ C}+0.01138\, \mathrm{ D}+0.000054\, {\mathrm{A}}^{2}-0.000074\, {\mathrm{B}}^{2}-0.000010\, {\mathrm{ C}}^{2}-0.000010\, {\mathrm{D}}^{2}-0.000010\, \mathrm{AB}-0.000020\, \mathrm{ AC}+0.000009\, \mathrm{ AD}+0.000006\, \mathrm{ BC}+0.000010\, \mathrm{ BD}+0.000004\, \mathrm{ CD}$$

Summaries of the developed regression expressions are displayed in Table 2, together with the individual desirability function values. As shown, values of linearity parameters (R^2^, R^2^-adjusted (R^2^-adj)) reveal that the obtained regression models are linear. Model predictability power was manifested by the elevated values of R^2^–predicted (R^2^–pred) and the low relative error (RE) values (Table S1, Supplementary Materials). The favorability of a certain variable blend that could be used to get the maximum response is reflected by the high value of the *d* – function (Derringer and Suich 1980, Myers et al. 2009).

**Table 2:** Summaries of the semi-empirical regression models for removing PRO and PMT from their individual solutions, Eqs. (6)– (9)

Dependent response	Model summary – Single solutions			Variables blend and individual desirability (*d*) – Single solutions	Variables blend and composite desirability (D) – Binary mixtures
	R^2^%	R^2^ – adj%	R^2^ – pred%		
%R_(PRO)_	99.22	98.38	95.56	AD = 108 mg/13 mL, [PRO] = 100 ppm, CT = 10 min, PyTemp = 700 °C (*d* = 1.0000, %R_(PRO)sin_ = 98.64%)	AD = 110 mg/13 mL, [DRUG] = 100 ppm, CT = 10 min, PyTemp = 700 °C (D = 1.0000, %R_(PRO)bin_ = 98.63%, %R_(PMT)bin_ = 94.14%)
%R_(PMT)_	99.01	97.96	94.00	AD = 120 mg/13 mL, [PMT] = 100 ppm, CT = 32 min, PyTemp = 700 °C (*d* = 1.0000, %R_(PMT)sin_ = 95.87%)	
*q*_*e* (PRO)_	99.98	99.95	99.84	AD = 20 mg/13 mL, [PRO] = 100 ppm, CT = 10 min, PyTemp = 700 °C (*d* = 1.0000, *q*_*e* (PRO)sin_ = 47.08 mg/g)	AD = 20 mg/13 mL, [DRUG] = 100 ppm, CT = 120 min, PyTemp = 700 °C (D = 1.0000, *q*_*e*(PRO)bin_ = 47.08 mg/g, *q*_*e*(PMT)bin_ = 39.84 mg/g)
*q*_*e* (PMT)_	99.95	99.89	99.72	AD = 20 mg/13 mL, [PMT] = 100 ppm, CT = 120 min, PyTemp = 700 °C (*d* = 1.0000, *q*_*e* (PMT)_ = 39.94 mg/g)	

#### Response optimization: individual drug solutions

Investigation of the response surface was performed by contemplating all the significant variable-variable interactions in the CCD to optimize the substantial variables and illustrated the nature of the response surface. Contour (2D) and surface (3D) plots were utilized therefore for this purpose. Sample contour plots are displayed in Fig. 1c, d for PRO and PMT, correspondingly. In Fig. 1c, %R_(PRO)_ was the response investigated, and a %R ˃ 90% is represented by the maroon color, while in Fig. 1d, %R_(PMT)_ was investigated, and a %R ˃ 85% is represented by dark grey color. These regions represent zones where the combined effect of both variables could achieve the maximum response. The findings of contour and surface plots were a good match with the *d-*values shown in Table 2.

#### Multi-response optimization: drug mixture

The composite desirability function (D) was utilized to determine the ideal parameters that simultaneously maximize each observed response for the binary mixtures. Table 2 shows the optimum parameters with a D-value of 1.0000 for both responses. Considering these optimum conditions, the removal efficiency of PRO and PMT simultaneously at 100 ppm [drug] are 98.63% and 94.14%, respectively. The highest adsorption capacity, *q*_*e*_ of PRO and PMT at 100 ppm [drug], are 47.08 mg/g and 39.48 mg/g, respectively.

#### Confirmatory experiments at optimum conditions

To further confirm the findings of the desirability plots listed in Table 2 for the uptake of PRO and PMT from the single and binary mixtures, 5 experimental runs were performed for each response employing the optimum conditions. Data (Table S3, Supplementary Materials) shows an average %R of 98.23% ± 0.38 for a 100 ppm PRO from its single solution, compared to 96.03% ± 0.45 in the case of PMT. For the same concentration of PRO and PMT in a binary mixture (100-ppm each), the %R was 97.43% ± 0.99 and 95.05% ± 0.41, respectively. The %error, compared to the values listed in Table 2, did not exceed 1.22%, confirming the high accuracy of the obtained response values. For *q*_*e*_, similar conclusions could be derived, where the %error did not exceed 4.22% (Table S3, Supplementary Materials).

### Adsorbent characterization

#### FT-IR analysis and point-of-zero-charge (pHPZC)

**Fig. 2:**
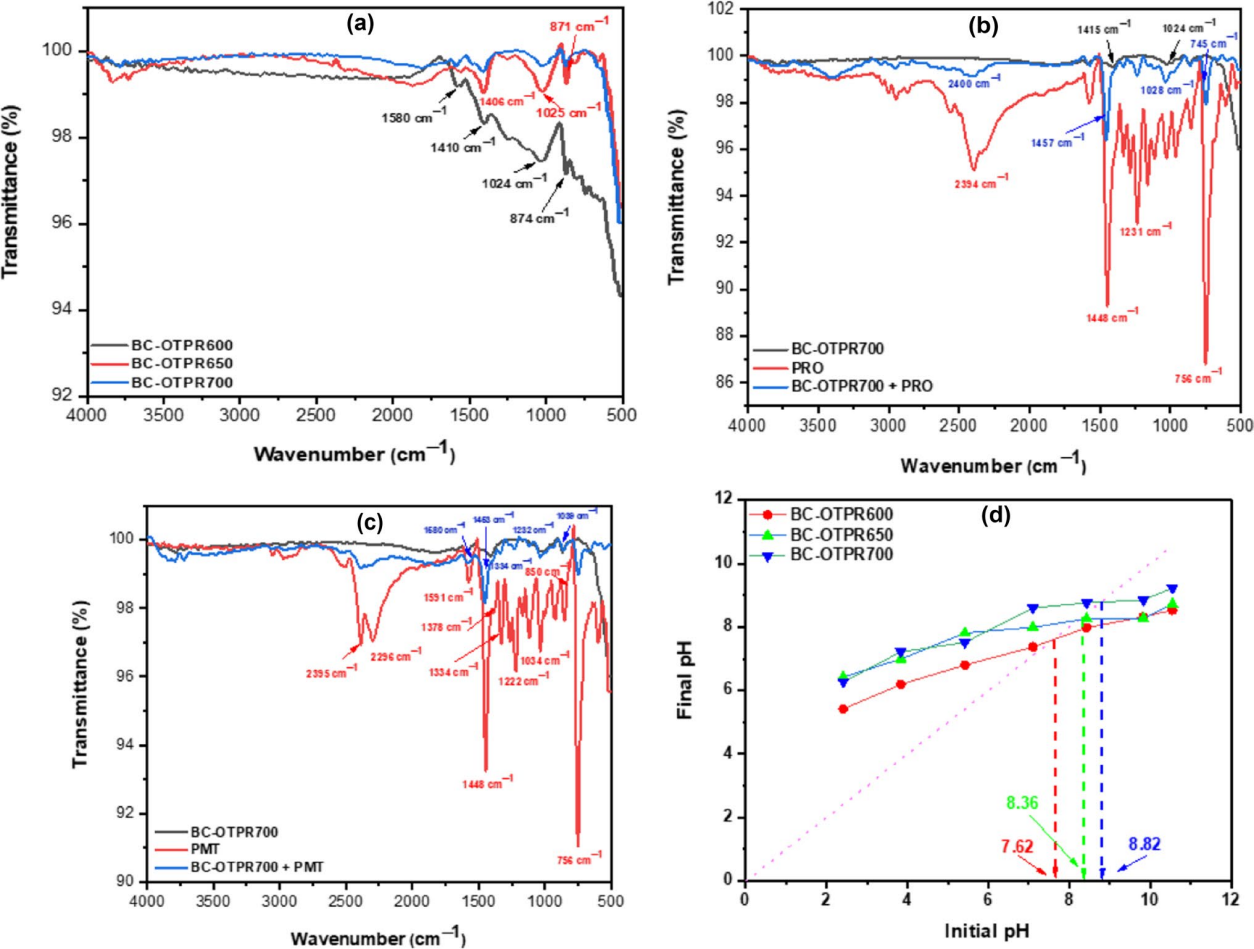
FT-IR spectra of (**a**) three adsorbents including BC-OTPR600, BC-OTPR650, and BC-OTPR700, (**b**) BC-OTPR700 before and after adsorption of PRO, (**c**) BC-OTPR700 before and after adsorption of PMT and (**d**) pH_PZC_ for the three adsorbents

FT-IR was used to explore the type of adsorption taking place. Spectra were recorded for the as-prepared adsorbents BC-OTPR600, BC-OTPR650, and BC-OTPR700, as well as for PRO and PMT before and after adsorption. The IR spectrum for the three adsorbents is shown in Fig. 2a. The spectrum of BC-OTPR600 shows some strong absorption bands which, under the effect of pyrolysis, either became of lower intensity (BC-OTPR650) or totally disappeared (BC-OTPR700). The absorption band at 1580 cm^−1^ was attributed to the N–H stretching vibration, a characteristic band for all lignocellulosic fibers. In addition, the cellulose C-H stretching could explain the band at 1410 cm^−1^. Furthermore, bands at 1024 and 874 cm^−1^ could correspondingly be assigned to C-O stretching and C = C bending of alkene. On the other hand, the FT-IR spectrum of PRO and PMT prior to and following the adsorption onto BC-OTPR700, respectively. The obtained data shows identical bands for both PRO and PMT (Fig. 2b, c), including the bands at 2395 and 2296 cm^−1^ related to NH^+^ stretching. Additionally, the absorption band at 1591 cm^−1^ could be ascribed to aromatic C = C stretching. The absorption band at 1448 cm^−1^ might be assigned to CH_3_ and CH_2_ bending. The bands at 1378 and 1334 cm^−1^ might correspond to the CH_3_ bending and C-N stretching of tertiary amines, respectively. Finally, the absorption bands between 850 – 860 cm^−1^ are assigned to the out-of-plane C-H bending of aromatic compounds (Banjare et al. 2021, Rajeswari and Kumari 2019).

The FT-IR spectrum of each drug, following its adsorption, clearly shows shifts and changes in the peak intensities. For instance, the absorption band at 1591 cm^−1^, which corresponds to C = C for PMT, has shifted to 1580 cm^−1^. Similarly, the band at 1378 cm^−1^ (C-N) has also shifted to 1334 cm^−1^. These findings suggest the probability of the formation of chemical bonds between these groups and the functional groups present on the surface of the biochar. The absorption band on the surface of the biochar at 1024 cm^−1^ (C-O) has also shifted to 1039 cm^−1^ due to the presence of such chemical bonding. The obtained data confirms the successful adsorption of both drugs onto the tested adsorbent. The point-of-zero-charge of the investigated adsorbents (Fig. 2d) and the data revealed that the pH_PZC_ was 7.62, 8.36, and 8.82 for BC-OTPR600, BC-OTPR650, and BC-OTPR700, respectively. Taking BC-OTPR700 as an example, the surface of the BC-OTPR700 will be negatively charged if the pH is ˃8.82, and it will be positively charged if the pH is ˂ 8.82. Therefore, at pH 7.00 ± 0.20, the adsorbent surface will be positively charged. Both PRO and PMT will also be positively charged, as reflected by their pK_a_ values (Domańska et al. 2011; Wilde et al. 2017). These findings suggest that the adsorption of PRO and PMT could be physisorption rather than chemisorption. The values of the adsorption energies, which are presented in the following sections, will provide additional confirmation of this result.

#### CHN analysis

Results of the CHN analysis for three adsorbents, BC-OTPR600, 650, and 700 indicate that the wt.% of nitrogen in the three adsorbents was almost identical. In contrast, C (wt%) slightly increased from 83.16% for BC-OTPR600 to 84.18% for BC-OTPR700. Furthermore, H% decreased from 1.84% in BC-OTPR600 to 1.24% for BC-OTPR700 due to hydrogen loss during the pyrolysis process.

#### Raman analysis

**Fig. 3:**
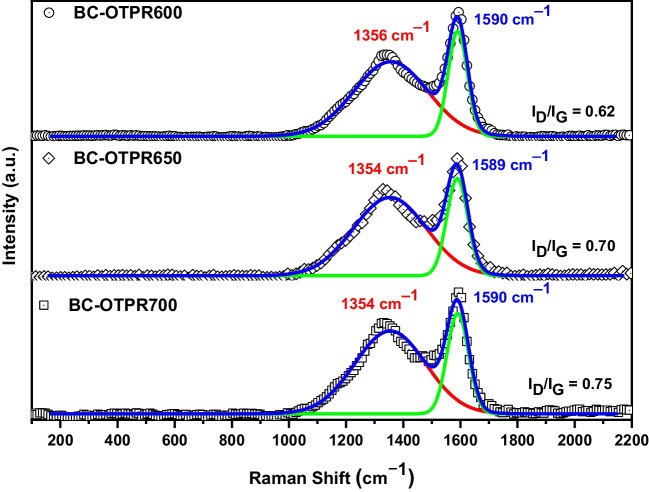
Raman spectra of the as-prepared samples BC-OTPR600, 650 and 700

Raman spectra of the as-prepared samples are displayed in Fig. 3. The obtained data shows the presence of two bands that are usually related to the carbonaceous materials: the band at ~ 1354 cm^−1^ (D-band) and ~ 1590 cm^−1^ (G- band). These two bands generally feature carbonaceous materials derived from thermally treated agro-wastes. In addition, the D-band corresponds to the carbon 3D features (defects and sizes), whilst the G-band perceives the C–C stretching for *sp*^*2*^ system. In addition, D-band intensity compared to the G-band (I_D_: I_G_) has increased from 0.62 for BC-OTPR600 to 0.75 for BC-OTPR700. The acquired data indicate that the defects on the surface of the BC-OTPR have grown as the pyrolysis temperature increased, and the presence of these defects on the surface has a positive effect on the adsorption efficiency for both PRO and PMT.

#### Textural analysis of the tested adsorbents

Brunauer–Emmett–Teller (BET) analysis was employed to determine the surface area, total pore volume, and average pore radius (PR) of the as-prepared adsorbents. The obtained data are presented, and the N_2_ adsorption–desorption isotherms are shown (Fig. S2 and Table S4, Supplementary Materials). Obtained data reveal that the Langmuir surface area (SA) has risen from 21.15 m^2^/g (BC-OTPR600) to 53.77 m^2^/g (BC-OTPR700). This behavior shows the positive impact of pyrolysis temperature on the surface area of the prepared biochar and, accordingly, its removal efficiency. In addition, the adsorption isotherm for the BC-OTPR600 was of type V, reflecting the significant effect of the intermolecular attraction and that adsorption could have occurred in pores and capillaries. On the other hand, the adsorption isotherm started to switch to type IV in BC-OTPR650, and it became entirely type IV in BC-OTPR700, indicating the presence of monolayer–multilayer adsorption and capillary condensation afterwards, an issue which significantly influences the adsorption efficiency of the prepared sorbents. In the same itinerary, the hysteresis loop for the three adsorbents was of the H3 type, which is commonly noticed in solids with a significant pore size distribution. Furthermore, all three adsorbents are composed of two types of pores; where ˃90% of pores are mesopores, which can be seen in the range between 2–50 nm, and a slight amount of macropores (> 50 nm) compared to the mesopores.

#### Morphological features of the prepared adsorbents: SEM and EDX analyses

**Fig. 4:**
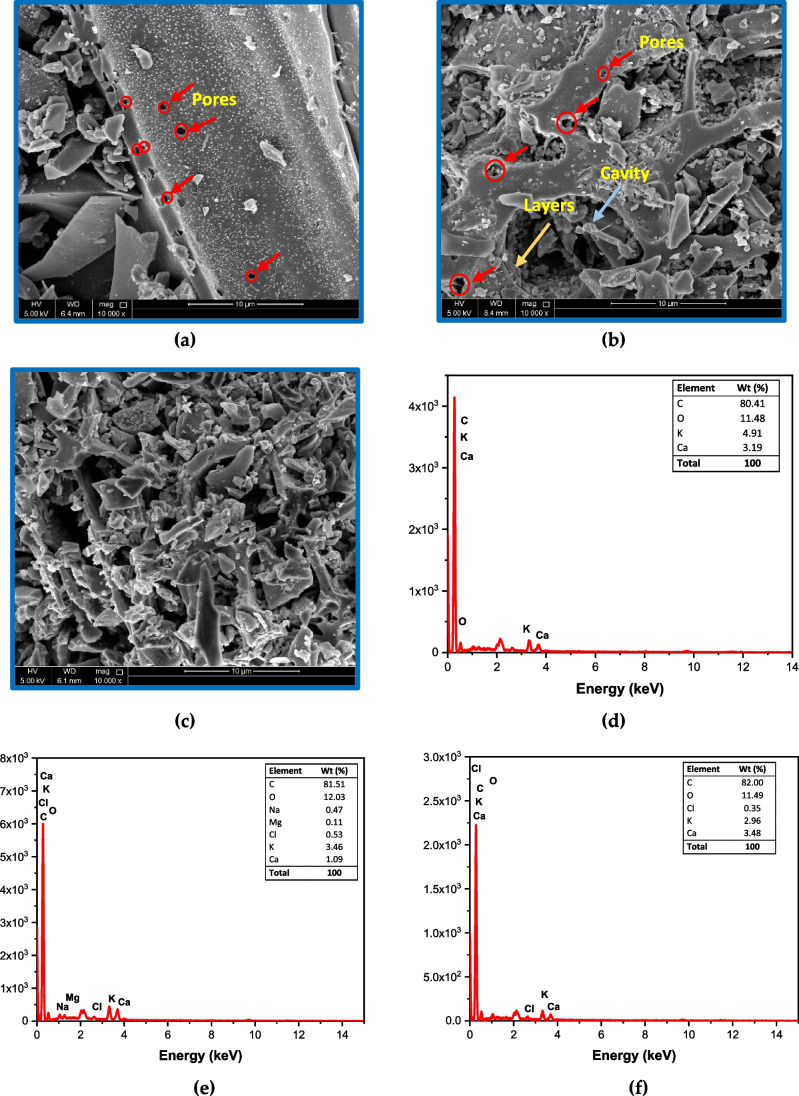
SEM micrographs of (**a**) BC-OTPR600, (**b**) BC-OTPR650, (**c**) BC-OTPR700 at 10 000 × magnifications, (**d**, **e**, **f**) EDX analyses of BC-OTPR600, 650 and 700, respectively

SEM–EDX analysis of the three adsorbents was performed (Fig. 4a-c). The micrograph of BC-OTPR600 shows the presence of a smooth surface with a small number of pores. On the other hand, the SEM micrograph for BC-OTPR650 reveals the occurrence of several mesopores and micropores that could have been formed under the effect of increasing the pyrolysis temperature. The existence of these pores was more prominent on the biochar surface of BC-OTPR700, which shows more cavities which in turn are reflected on the surface area and hence the adsorption efficiency of the BC-OTPR700.

EDX analysis, Fig. 4d-f shows that the % surface carbon has increased by increasing the pyrolysis temperature, from 80.41% at 600 °C to 82.00% at 700 °C. On the other hand, the %oxygen was not much affected by the pyrolysis process. Moreover, the potassium concentration decreased from 4.91% (BC-OTPR600) to 2.69% (BC-OTPR700).

### Adsorption isotherms and kinetic investigations

#### Adsorption isotherms

Most studies on drug adsorption by different adsorbents have aimed at the depollution of PhACs from their individual solutions. In real circumstances, however, PhACs do not exist isolated; instead, as a multi-component mixture. Another important fact is that the adsorption equilibrium modelling of a multi-component system, which is crucial when designing treatment systems, is usually overlooked. One of the significant difficulties identified in this study is the structural similarity of PRO and PMT. The competition between both drugs to occupy the adsorption sites is another crucial aspect that needs to be comprehended. Adsorption equilibrium isotherms such as extended Langmuir are commonly used to comprehend the competitive adsorption conduct of the various molecules in a binary system (Febrianto et al. 2009, Foo and Hameed 2010, Kumar et al. 2008b, Zolgharnein et al. 2015). The adsorbate-adsorbent interaction and adsorbate accrual on the sorbent surface may well be estimated utilizing the adsorption isotherms. Adsorption of PRO and PMT, onto BC-OTPR700 was studied using four well-established models: Langmuir, Freundlich, Temkin, and Dubinin–Radushkevich (D–R) (Araújo et al. 2018; Kumar et al. 2008a; Langmuir 1918); in the case of a single drug solution, in addition to extended Langmuir isotherm for the binary mixture (Moussavi and Barikbin 2010, Zolgharnein et al. 2015).

**Fig. 5:**
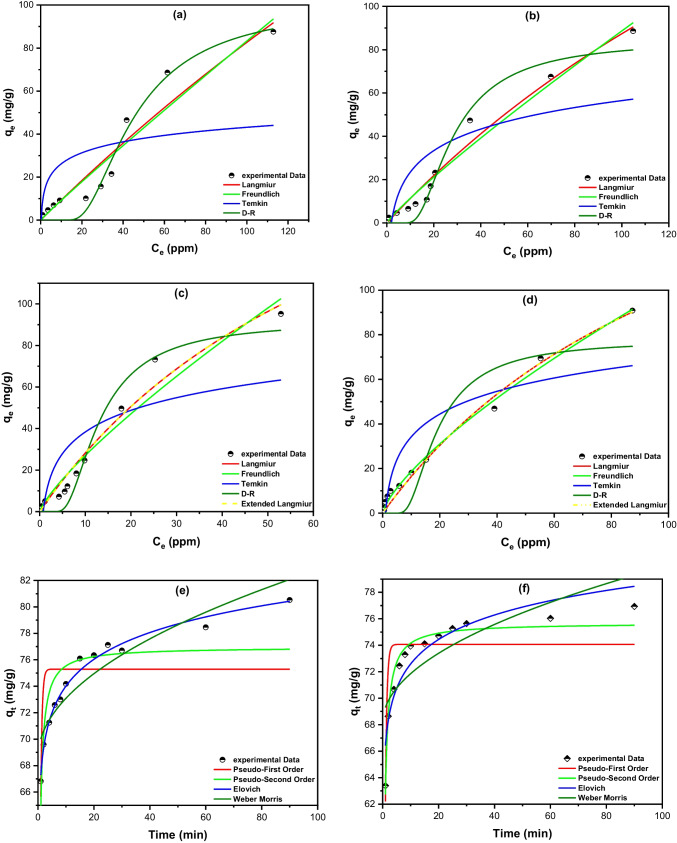
Adsorption isotherms of BC-OTPR700 adsorbent with (**a**) PRO_(sin)_, (**b**) PMT_(sin)_, (**c**) PRO_(bin)_, (**d**) PMT_(bin)_ and the kinetic models for the adsorption of (e)PRO and (f) PMT onto BC-OTPR700

Langmuir model is based on three hypotheses: 1) the Energy of the existing adsorption sites is equal; 2) Every adsorbate molecule dominates one sorption site on the surface without the interactions between the adsorbed species, and 3) The adsorption is confined mostly on the adsorbent surface. Langmuir isotherm is illustrated by Eq. (10) and Fig. 5a, b for PRO and PMT in single solutions, in turn, and Fig. 5c, d for the binary mixture.

10 $${q}_{e}=\frac{{q}_{m} {K}_{L} {C}_{e}}{1+{K}_{L} {C}_{e}}$$

In this equation, *q*_*m*_ and *K*_*L*_ represent the maximum adsorption capacity, and Langmuir equilibrium coefficient, respectively. Langmuir model could be represented by the dimensionless form depicted by Eq. (11):

11 $${R}_{L}=\frac{1}{1+{K}_{L} {C}_{0}}$$

where *R*_*L*_ and *C*_*0*_ (ppm) signify the separation factor and initial concentrations of PRO and PMT, respectively, the adsorption desirability could be determined considering the *R*_*L*_ value. Thus, the process is ‘spontaneous’ if 0 ˂ *R*_*L*_ ˂ 1. If *R*_*L*_ value, however, exceeds 1, the adsorption process is ‘unfavorable’. On the other hand, If *R*_*L*_ = 1, the adsorption is linear, and if *R*_*L*_ = 0, the adsorption process is irreversible Table 3. The obtained *R*_*L*_ values for the adsorption of PRO and PMT onto BC-OTPR700 from their single and binary solutions were found to be ˂1 in either case, inferring that the adsorption was spontaneous. Moreover, the obtained data shows that the adsorption becomes irreversible at higher concentrations of both PRO and PMT, with a maximum adsorption capacity (*q*_*m*_) of 640.76 and 346.95 mg/g from their single solutions, respectively. A comparison between the adsorption capacity of BC-OTPR700 and various adsorbents for the removal of organic and inorganic pollutants is exhibited in Table 4. Attained data shows the superiority of the current adsorbent for the remediation of PRO and PMT.

**Table 3:** Non-linear equations of the four equilibrium isotherms were adopted for sorption of PRO and PMT onto BC-OTPR700 from single solution (sin) and binary mixture (bin)

Langmuir	*q*_*m*_ (mg/g)	*K*_*L*_ (L. mole^−1^)	R^2^	
PRO_(sin)_	640.76	0.0018	0.9248	
PMT_(sin)_	346.95	0.0034	0.9733	
PRO_(bin)_	243.01	0.0131	0.9665	
PMT_(bin)_	216.48	0.0082	0.9882	
Freundlich	1/*n*	*K*_*F*_ (mole/g) (L/mole)^1*/n*^	R^2^	
PRO_(sin)_	0.96	1.016	0.9217	
PMT_(sin)_	0.89	1.469	0.9676	
PRO_(bin)_	0.80	4.282	0.9485	
PMT_(bin)_	0.73	3.447	0.9942	
Temkin	*b*_*T*_ (J/mole)	*A*_*T*_ (L/mole)	R^2^	
PRO_(sin)_	336.35	3.503	0.4060	
PMT_(sin)_	172.63	0.513	0.6337	
PRO_(bin)_	165.01	1.287	0.6461	
PMT_(bin)_	168.19	1.019	0.8063	
D–R	$\beta$	*E* (kJ/mole)	*q*_*m*_ (mg/g)	R^2^
PRO_(sin)_	5.06 × 10^–7^	0.99	100.39	0.9696
PMT_(sin)_	2.02 × 10^–7^	1.57	84.49	0.9685
PRO_(bin)_	4.48 × 10^–8^	3.34	91.61	0.9572
PMT_(bin)_	9.26 × 10^–8^	2.32	77.63	0.8822
Extended-Langmuir	*q*_*m*_ (mg/g)	*K*_*L(PRO)*_ (L. mole^−1^)	*K*_*L(PMT)*_ (L. mole^−1^)	R^2^
PRO_(bin)_	360.49	8.9621	13.296	0.9665
PMT_(bin)_	185.54	3.8992	3.3412	0.9882

**Table 4:** Adsorption capacities of different adsorbents compared to BC-OTPR700

Adsorbent	Adsorbate	Adsorption capacity (*q*_*max,*_ mg/g)	Reference
Magnetic activated carbon nanocomposite	Promazine	101.01	(D'Cruz et al. 2020a)
Carbonaceous/spinel ferrite nanocomposite	Promazine	90.91	(Al-Hetlani et al. 2022b)
Olive stone and olive tree pruning derived-activated carbons	Phenolic compounds	188.6 – 359.0	(Esteves et al. 2022)
Olive stone-derived activated carbon	Methylene blue	16.10	(Hazzaa and Hussein 2015)
Olive stone-derived activated carbon	Hydroxytyrosol	375.0	(Eder et al. 2021)
Olive stone derived KOH modified activated carbons	Methylene blue	263	(Stavropoulos and Zabaniotou 2005)
Chemically modified olive tree pruning activated carbons	Lead (II)	27.05 (untreated)–121.6 (NaOH modified)	(Calero et al. 2013b)
Chemically activated olive tree pruning	Lead (II)	84.03 (sulfuric acid modified)–121.9 (NaOH modified)	(Calero et al. 2013c)
Chemically treated olive tree pruning	Lead (II)	12.97 (untreated)–16.04 (NaOH modified)	(Ronda et al. 2013)
BC-OTPR700	Promazine	(sin) 640.76 (bin) 243.01	Current study
	Promethazine	(sin) 346.95 (bin) 216.48	

On the other hand, the value of *q*_*m*_ in the binary mixture was 243.01 mg/g for PRO and 216.48 mg/g for PMT. The decrease in *q*_*m*_ in the case of a binary mixture could be attributed to the rivalry of PRO and PMT to occupy the sorption sites. These data are consistent with the findings of the desirability functions obtained from the CCD, indicating that BC-OTPR700 has a better adsorption capacity towards PRO compared to PMT in single systems. This favorability extends to the binary mixture at almost all concentrations (100 ppm – optimum [Drug] as per the CCD outputs, and up to 800 ppm as used in Langmuir isotherm). In the same itinerary, the change in *q*_*m*_ from single to binary systems is trivial, implying that the competition between PRO and PMT towards sorption sites at low concentrations is insignificant.

The Freundlich isotherm model, unlike Langmuir, expresses multiple layers of sorption with a heterogeneous energy allocation through heterogeneous surfaces, Eq. (12):

12 $${q}_{e}= {K}_{F}{C}_{e}^\frac{1}{n}$$

In this equation, *K*_*F*_ (mole.g^−1^)(L.mole^−1^), and 1/*n* is the Freundlich coefficient that illustrates the adsorbent capacity and the difference in the adsorption intensity, as well as the aberration from linearity. The data acquired for PRO and PMT in a single solution and a binary mixture and the Freundlich isotherm calculated parameters are shown in Fig. 5 and Table 3. The attained data for single solutions shows that the adsorption of PRO in both systems best conforms to the Langmuir isotherm. Howbeit, adsorption of PMT_(sin)_ can be better illustrated by Langmuir isotherm compared to the sorption of PMT_(bin),_ which conforms to the Freundlich isotherm. Data shown in Table 3 reveals that PRO_(sin)_ has a 1/*n* = 0.96, *n* = 1.05 while PMT_(sin)_ has a 1/*n* = 0.89, *n* = 1.12. Therefore, the adsorption potential (A = *n*RT) for PRO_(sin)_ is 2.15 kJ and for PRO_(sin)_ 2.29 kJ, implying that any drug molecule with potential energy less than these values will be adsorbed onto the BC-OTPR700 surface, and the process considered as favorable and irreversible. The value of 1/*n* in the binary mixture was 0.80 and 0.73 for PRO_(bin)_ and PMT_(bin)_, respectively, indicating favorable and irreversible adsorption of the two drugs from a binary mixture.

Temkin isotherm is utilized to depict the adsorbate-adsorbent interactions; consequently, the sorption heat of the drug molecules present in a layer onto the studied adsorbent will gradually decrease in the presence of such interaction, as revealed by Eq. (13)

13 $${q}_{e} = \frac{RT}{{b}_{T}}\mathrm{ln}({ {A}_{T}C}_{e })$$

*A*_*T*_ represents the Temkin isotherm constant, *R* represents the universal gas constant (8.314 J/mol K), *b*_*T*_ is known as the constant of the Temkin isotherm, and *T* represents the temperature (K). According to the findings in Fig. 5a-d and Table 3, PRO_(sin)_ has a sorption energy of 336.35 J/mol, compared to 172.63 J/mol in the case of PMT_(sin)_. The obtained data indicate that PRO and PMT adsorb favorably on BC-OTPR700 in a single solution and confirm the resulting data from the previous equilibrium isotherms. The same results were obtained for the binary system, with sorption energies of 165.01 and 168.19 J/mol for PRO and PMT, respectively.

The D–R equilibrium isotherm was employed to propose the sorption mechanism, and it can be represented by Eq. (14):

14 $${q}_{e}= {q}_{s} \mathrm{exp}\left(-\beta . {\epsilon }^{2}\right)$$

where *q*_*s*_ is the saturation capacity, *β* represents the activity coefficient, and it is used to find the drug sorption energy; E (kJ/mol), and *ϵ* is the calculated Polanyi potential. Equations (15) and (16) calculate *ϵ* and E, respectively, as follows:

15 $$\epsilon = RT(1+\frac{1}{{C}_{e}})$$

16 $$E= {~}^{1}\!\left/ \!{~}_{\sqrt{2\beta }}\right.$$

From the data given in Table 3, the obtained results for single solutions indicate that the sorption energy for PRO_(sin)_ is 0.99 kJ/mol compared to1.57 kJ/mol for PMT_(sin)_, suggesting physisorption of PRO and PMT onto BC-OTPR700 where the sorption energy is ˂7 kJ/mol. Comparable behavior was obtained for the two drugs in the binary mixture. The obtained data indicate that the adsorption PRO and PMT from single and binary systems depend mainly on the surface area of the BC-OTPR700 adsorbent.

Finally, the extended Langmuir isotherm was used to study the adsorption of the two drugs from their binary mixture, and it can be calculated by Eqs. (17) and (18):

17 $${q}_{e(PRO)}=\frac{{q}_{m(PRO)} {K}_{L(PRO)} {C}_{e (PRO)}}{1+{K}_{L(PRO)} {C}_{e(PRO)}{+K}_{L(PMT)} {C}_{e(PMT)}}$$

18 $${q}_{e (PMT)}=\frac{{q}_{m(PMT)} {K}_{L(PMT)} {C}_{e(PMT)}}{1+{K}_{L(PRO)} {C}_{e(PRO)}{+K}_{L(PMT)} {C}_{e(PMT)}}$$

The calculated parameters shown in Table 3 were determined through non-linear regression. R^2^ values of 0.9665 and 0.9882 were obtained for PRO_(bin)_ and PMT_(bin)_, respectively, revealing that the extended Langmuir isotherm is appropriate for representing the adsorption of PRO and PMT from a binary mixture. The *q*_*max*_ of BC-OTPR700 towards PRO and PMT in a multi-component system decreased, revealing the competition between the two drugs. According to Table 3, the maximum adsorption capacity for PRO_(bin)_ was 360.49 mg/g compared to 185.54 mg/g for PMT_(bin)_. These findings confirm the competitive adsorption between the two drugs in the binary mixture.

#### Kinetic studies

The adsorption kinetics of PRO and PMT onto BC-OTPR700 were investigated using four kinetic models: pseudo-first-order (PFO), pseudo-second-order (PSO), Elovich, and Weber–Morris (WM) (Ezzati 2020; Issa et al. 2014; Wu et al. 2009). Data shown in Fig. 5e, f display the relationship between the amount of adsorbed drug, *q*_*t*_ (mg/g), and time (min) for PRO and PMT adsorption onto BC-OTPR700, respectively. The measured parameters for these four models are listed in Table 5. The obtained data indicate that the pseudo-second-order (PSO) model (R^2^ = 0.7746 for PRO and 0.9628 for PMT) could be used to describe the adsorption of both drugs onto BC-OTPR700. These findings imply that the concentration of drug and adsorbent impacts the rate of the adsorption reaction and can be represented by Eq. (19) as follows:

**Table 5:** Calculated parameters for four kinetic models, including pseudo-first-order (PFO), pseudo-second-order (PSO), Elovich, and Weber–Morris (WM)

Models	Parameter	Value	
		PRO	PMT
Pseudo-first order (PFO) $\frac{d{q}_{t}}{dt}$= *k*_*1*_*(q*_*e−*_*q*_*t*_*)*	K_1_ (min^−1^)	2.054	1.832
	*q*_*e*_ (mg/g)	75.29	74.07
	R^2^	0.4337	0.7188
Pseudo-second order (PSO) $\frac{d{q}_{t}}{dt}$= *k*_*2*_*(q*_*e−*_*q*_*t*_*)*^*2*^	K_2_ *(*g.mg^−1^.min^−1^*)*	0.069	0.064
	*q*_*e*_(mg/g)	76.95	75.68
	R^2^	0.7746	0.9628
Elovich model *q*_*t*_ = $\frac{1}{\beta } \times ln(1+\alpha \beta t)$	α	3.00 × 10^10^	1.67 × 10^11^
	β	0.34	0.374
	R^2^	0.9855	0.8794
Weber − Morris model (WM) ${q}_{t}={K}_{I}{t}^{0.5}+C$	K_I_	1.420	1.177
	C	68.60	68.13
	R^2^	0.8542	0.6245

where K_2_ is rate constant (g.mg^−1^.min^−1^) and q_t_ is adsorbed quantity at time t; while α and β are initial sorption concentration rate (mg.g^−1^.min^−1^), and desorption constant (g/mg), K_I_ is intraparticle diffusion rate constant (mg.g^−1^.min^−0.5^), and C is boundary thickness effect

19 $$\mathrm{PRO }\&\mathrm{ PMT}+\mathrm{BC}-\mathrm{OTPR}700 \left(\stackrel{\mathrm{k}}{\to } \right) \left\{\mathrm{PRO}@\mathrm{BC}-\mathrm{OTPR}700\right\}\mathrm{or }\left\{\mathrm{PMT}@\mathrm{BC}-\mathrm{OTPR}700\right\}$$

The Elovich model reveals substantial initial adsorption (α) (α for PRO = 3 × 10^10^ mg.g^−1^.min^−1^ and for the PMT = 1.67 × 10^11^ mg.g^−1^.min^−1^). In addition, the R^2^ value was high in the case of PRO (0.9855) compared to the other models; therefore, the Elovich model can be utilized to depict the adsorption of PRO. Finally, Weber–Morris (WM) cannot be used to explain the adsorption of both drugs onto BC-OTPR700, where the R^2^ value was too low for both drugs compared to other kinetic models.

### Proposed adsorption mechanism

Electrostatic interactions, π-π interactions, hydrogen bonding, pore filling, and surface diffusions might be used to explain the adsorption of PRO and PMT onto BC-OPPR700 adsorbent.

**Fig. 6:**
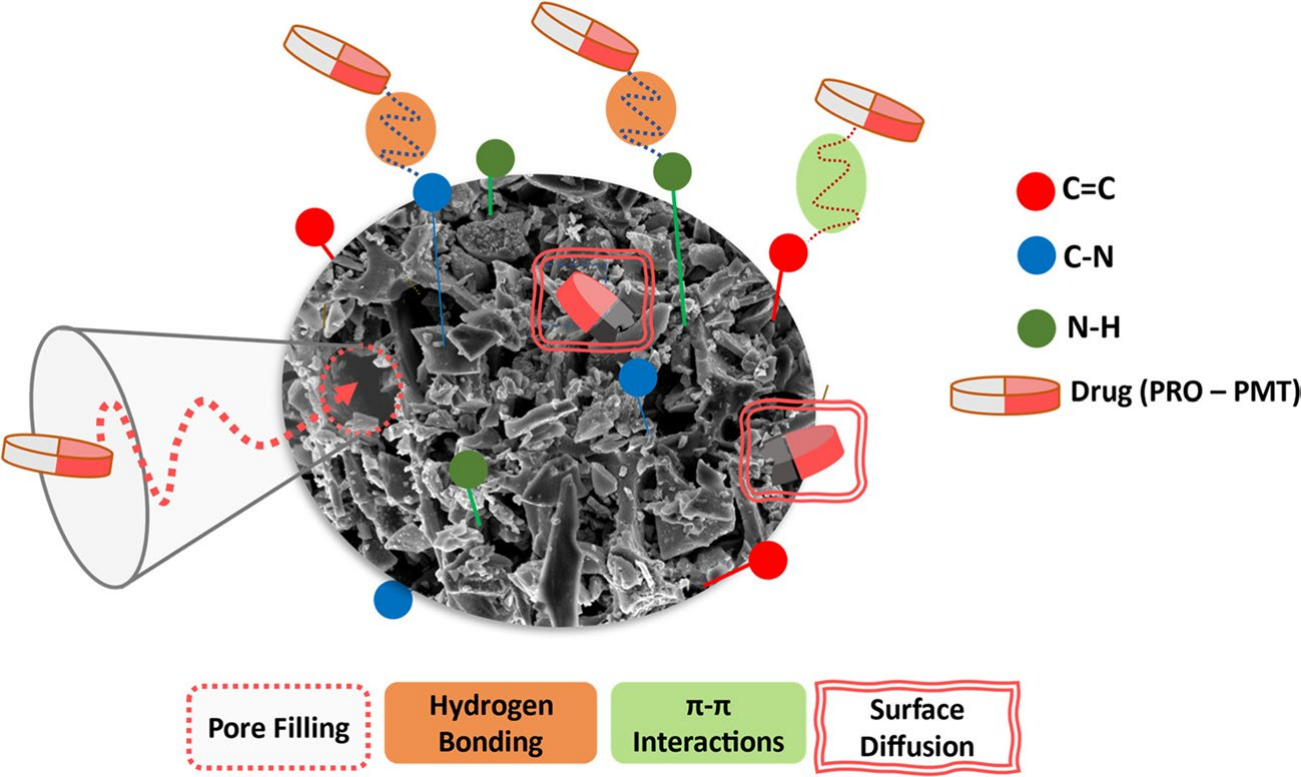
Proposed mechanisms for the adsorption interaction between the two drugs; PRO and PMT, onto the as-prepared adsorbent, BC-OTPR700

1. Electrostatic interactions occur when the charged functional groups of PRO and PMT interact with the charged surface of BC-OTPR700. The pH of the solution and the pH_PZC_ are the two most important factors that impact the electrostatic interactions. In the current investigation, the pH of the solution is 7.0, which is less than the pK_a_ of both PRO and PMT and less than the pH_pzc_ of the studied adsorbent. This results in positively charged drug molecules and adsorbent surface, an issue which negatively affects the electrostatic interaction. Hence, the adsorption process will mainly occur via the physisorption pathway.

2. π-π interactions and hydrogen bonding: the biochar surface is rich in functionalities such as C = C and could have electron-rich or electron-deficient properties that facilitate the interaction, Fig. 6, (Shin et al. 2022; Yao et al. 2010). In addition, there may be hydrogen-bonding interactions between the oxygen atoms (H-acceptors) in BC-OTPR700 and the hydrogen of the amine groups (H-donors) on both PRO and PMT drugs. This type of interaction is commonly referred to as hydrogen bonding between dipoles.

3. Pore-filling and surface diffusions mechanisms: intra-particle diffusion is the movement of adsorbate molecules into the pores of the adsorbent surface, known as the pore-filling effect (Shin et al. 2022; Xiang et al. 2020). In the case of BC-OTPR700, SEM and BET analyses confirmed the existence of many pores on the surface, and both drugs (PRO and PMT) diffused into the pores of the adsorbent and interacted with the BC-OTPR700 surface. Surface diffusion, on the other hand, refers to the movement of adsorbate molecules on the surface of the BC-OTPR adsorbent. The drug molecules diffuse along the surface of the as-prepared biochar and interact with the surface functional groups. Both mechanisms are essential for describing the adsorption of PRO and PMT onto BC-OTPR8700, thus increasing the interaction between the two drugs and the adsorbent surface, and enhance the adsorption process greatly.

### Desorption and recovery studies

**Fig. 7:**
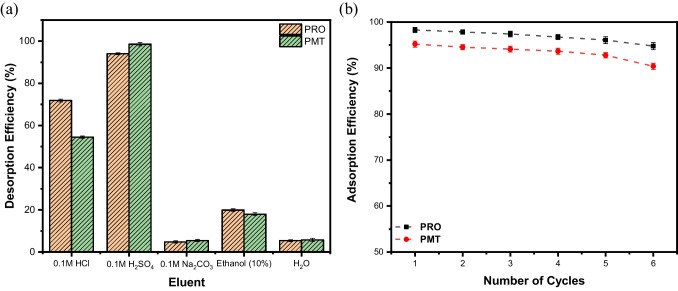
**a** Effect of different eluents on desorption of both PRO and PMT from loaded BC-OTPR700, and **b** regeneration studies of the studied adsorbent BC-OTPR700 towards the removal of both PRO and PMT

The economic value of any adsorbent is crucial and largely depends on its ability to be regenerated. A desorption investigation study was conducted for this purpose using five eluents, followed by six sequential adsorption–desorption cycles. Figure 7a depicts the relationship between the tested eluents and the desorption efficiency (%). Results showed that 0.1 M sulfuric acid was the most effective eluent for both PRO and PMT, achieving desorption efficiencies of 94.06% and 98.54%, correspondingly. Therefore, 0.1 M sulfuric acid was selected as the preferred eluent for desorbing PRO and PMT from the drug-loaded BC-OTPR700.

To study the regeneration of the adsorbent, cyclic adsorption–desorption experiments were conducted, and the outcomes are depicted in Fig. 7b. The obtained results demonstrate that the efficiency of PRO removal using BC-OTPR700 adsorbent experienced a slight reduction from 98.27% in cycle 1 to 94.75% in cycle 6. Similarly, PMT adsorption efficiency also decreased slightly from 95.19% in cycle 1 to 90.37% in cycle 6. These results indicate that the examined adsorbent is stable and can be effectively regenerated and reused for over six cycles, maintaining more than 90% removal efficiency.

## Conclusions

This work is the first to describe the use of OTPR to depollute PRO and PMT from their single and binary systems. Central composite design (CCD) was used to maximize %R and *q*_*e*_ via maneuvering of four variables. BC-OTPR700 showed excellent removal efficiency for PRO and PMT from single solutions (98.64 and 95.87%, respectively). D-R isotherm revealed that the adsorption of PRO and PMT from a single solution and binary mixture was physisorption, implying the formation of drug multilayers on the surface of BC-OTPR700. Kinetic experiments demonstrated that the sorption of PRO and PMT onto BC-OTPR700 conformed to a pseudo-second-order model.

## Supplementary information

Below is the link to the electronic supplementary material.

Supplementary file1 (DOCX 160 KB)

## Data Availability

The data discussed in the manuscript are included in the text itself or in the supplementary material.
