# Supplementary material for: Competitive adsorptive removal of promazine and promethazine from wastewater using olive tree pruning biochar: operational parameters, kinetics, and equilibrium investigations
Source: Environ Sci Pollut Res Int. 2023 Jun 16;30(34):82387–405. doi: 10.1007/s11356-023-27688-6 (PMC10349721; doi:10.1007/s11356-023-27688-6)
Supplement: Supplementary file 1 — Supplementary file1 (DOCX 160 KB) [file 11356_2023_27688_MOESM1_ESM.docx]

*Supplementary information for*

**Competitive Adsorptive Removal of Promazine and Promethazine from Wastewater Using Olive Tree Pruning Biochar: Operational Parameters, Kinetics, and Equilibrium Investigations**

Marwa El-Azazy ^a♣^, Ahmed S. El-Shafie ^a^, Samer Fawzy ^b^, David W. Rooney ^b^, Ahmed I. Osman ^b, c*♣^

^a^ Department of Chemistry and Earth Sciences, College of Arts and Sciences, Qatar University, Doha 2713, Qatar.

^b^ School of Chemistry and Chemical Engineering, Queen’s University Belfast, Belfast BT9 5AG, Northern Ireland, UK

^c^ Chemistry Department, Faculty of Science, South Valley University, Qena 83523 – Egypt.

^♣ Equal contribution^

Corresponding Authors: Ahmed I. Osman, Email: [aosmanahmed01@qub.ac.uk](mailto:aosmanahmed01@qub.ac.uk)

Address: School of Chemistry and Chemical Engineering, Queen's University Belfast, David Keir Building, Stranmillis Road, Belfast BT9 5AG, Northern Ireland, United Kingdom

Fax: +44 2890 97 4687, Tel.: +44 2890 97 4412

**Fig. S1.** Absorption spectrum of 80 ppm solutions of PRO (λ_max_ = 300 nm), PMT (λ_max_ = 295 nm), and PRO-PMT binary mixture.

**Fig. S2.** Nitrogen adsorption-desorption isotherms for BC-OTPR600, 650 and 700, including surface area, SA (a), and the pore diameter, PR (b).

**Table S1.** Experimental scenario for the removal of PRO and PMT based on the CCD. Experimental values for the dependent responses: %R and *q_e_* and the relative error (RE) are shown.

| **Run#** | **Blk^1^** |  | **Variables and their codes** | | | **PRO** | | | | | | **PMT** | | | | | | |  |
| --- | --- | --- | --- | --- | --- | --- | --- | --- | --- | --- | --- | --- | --- | --- | --- | --- | --- | --- | --- |
|  |  | **A – AD** | **B -[Drug]** | **C – CT** | **D – PyTemp** | **%R_exp_^2^** | **%R_prd_^3^** | **RE^4^** | ***q_e exp_*^2^** | ***q_e prd_*^3^** | **RE^4^** | | **%R_exp_^2^** | **%R_prd_^3^** | **RE^4^** | ***q_e exp_*^2^** | ***q_e prd_*^3^** | **RE^4^** | |
| 01 | 1 | 20 (−) | 10 (−) | 10 (−) | 700 (+) | 43.59 | 54.03 | 0.19 | 3.37 | 3.73 | 0.10 | | 54.37 | 56.07 | 0.03 | 3.53 | 3.52 | 0.00 | |
| 02 | 1 | 120 (+) | 100 (+) | 10 (−) | 700 (+) | 97.11 | 98.25 | 0.01 | 10.52 | 10.77 | 0.02 | | 93.98 | 94.92 | 0.01 | 10.18 | 10.15 | 0.00 | |
| 03 | 1 | 120 (+) | 10 (−) | 10 (−) | 700 (+) | 24.21 | 41.92 | 0.42 | 0.30 | 0.35 | 0.14 | | 66.38 | 64.21 | 0.03 | 0.72 | 0.81 | 0.11 | |
| 04 | 1 | 120 (+) | 10 (−) | 120 (+) | 600 (−) | 78.03 | 81.39 | 0.04 | 4.00 | 4.34 | 0.08 | | 39.23 | 39.72 | 0.01 | 0.43 | 0.44 | 0.03 | |
| 05 | 1 | 70 (0) | 55 (0) | 65 (0) | 650 (0) | 69.03 | 73.05 | 0.06 | 7.05 | 7.41 | 0.05 | | 78.52 | 77.98 | 0.01 | 8.02 | 8.06 | 0.00 | |
| 06 | 1 | 120 (+) | 100 (+) | 120 (+) | 600 (−) | 77.53 | 78.90 | 0.02 | 7.97 | 8.03 | 0.01 | | 63.38 | 62.15 | 0.02 | 6.86 | 7.05 | 0.03 | |
| 07 | 1 | 70 (0) | 55 (0) | 65 (0) | 650 (0) | 70.86 | 73.05 | 0.03 | 7.24 | 7.41 | 0.02 | | 77.64 | 77.98 | 0.00 | 7.93 | 8.06 | 0.02 | |
| 08 | 1 | 70 (0) | 55 (0) | 65 (0) | 650 (0) | 70.05 | 73.05 | 0.04 | 7.16 | 7.41 | 0.03 | | 75.26 | 77.98 | 0.03 | 8.20 | 8.06 | 0.02 | |
| 09 | 1 | 70 (0) | 55 (0) | 65 (0) | 650 (0) | 69.33 | 73.05 | 0.05 | 7.08 | 7.41 | 0.04 | | 76.20 | 77.98 | 0.02 | 7.60 | 8.06 | 0.06 | |
| 10 | 1 | 20 (−) | 100 (+) | 120 (+) | 700 (+) | 76.97 | 78.82 | 0.02 | 46.03 | 47.08 | 0.02 | | 68.08 | 67.35 | 0.01 | 44.26 | 39.67 | 0.12 | |
| 11 | 1 | 20 (−) | 10 (−) | 10 (−) | 600 (−) | 77.36 | 79.20 | 0.02 | 5.03 | 5.29 | 0.05 | | 66.07 | 67.34 | 0.02 | 4.29 | 4.38 | 0.02 | |
| 12 | 1 | 120 (+) | 10 (−) | 120 (+) | 700 (+) | 48.92 | 54.72 | 0.11 | 0.44 | 0.53 | 0.17 | | 56.75 | 59.24 | 0.04 | 0.62 | 0.59 | 0.04 | |
| 13 | 1 | 120 (+) | 10 (−) | 10 (−) | 600 (−) | 70.95 | 75.10 | 0.06 | 4.20 | 4.31 | 0.03 | | 49.31 | 50.63 | 0.03 | 0.54 | 0.76 | 0.30 | |
| 14 | 1 | 20 (−) | 10 (−) | 120 (+) | 600 (−) | 94.74 | 97.18 | 0.03 | 6.13 | 6.46 | 0.05 | | 86.09 | 85.25 | 0.01 | 5.61 | 5.77 | 0.03 | |
| 15 | 1 | 20 (−) | 10 (−) | 120 (+) | 700 (+) | 80.14 | 82.09 | 0.02 | 5.21 | 5.30 | 0.02 | | 79.89 | 79.05 | 0.01 | 5.17 | 5.32 | 0.03 | |
| 16 | 1 | 20 (−) | 100 (+) | 120 (+) | 600 (−) | 56.57 | 61.85 | 0.09 | 29.06 | 29.81 | 0.03 | | 56.85 | 59.45 | 0.04 | 36.74 | 36.25 | 0.01 | |
| 17 | 1 | 20 (−) | 100 (+) | 10 (−) | 700 (+) | 70.36 | 73.64 | 0.04 | 45.74 | 46.11 | 0.01 | | 45.10 | 44.87 | 0.01 | 29.34 | 27.84 | 0.05 | |
| 18 | 1 | 120 (+) | 100 (+) | 10 (−) | 600 (−) | 84.53 | 87.97 | 0.04 | 9.19 | 9.76 | 0.06 | | 72.66 | 73.69 | 0.01 | 7.87 | 8.03 | 0.02 | |
| 19 | 1 | 120 (+) | 100 (+) | 120 (+) | 700 (+) | 86.73 | 90.19 | 0.04 | 9.40 | 9.73 | 0.03 | | 89.25 | 88.4 | 0.01 | 9.67 | 9.95 | 0.03 | |
| 20 | 1 | 20 (−) | 100 (+) | 10 (−) | 600 (−) | 40.74 | 49.43 | 0.18 | 29.98 | 30.43 | 0.01 | | 40.49 | 37.2 | 0.09 | 26.43 | 27.33 | 0.03 | |
| 21 | 2 | 70 (0) | 10 (−) | 65 (0) | 650 (0) | 83.95 | 79.94 | 0.05 | 1.56 | 1.37 | 0.14 | | 80.87 | 79.28 | 0.02 | 1.50 | 1.46 | 0.03 | |
| 22 | 2 | 70 (0) | 55 (0) | 120 (+) | 650 (0) | 81.50 | 79.33 | 0.03 | 8.32 | 7.94 | 0.05 | | 76.65 | 76.98 | 0.00 | 7.83 | 7.96 | 0.02 | |
| 23 | 2 | 70 (0) | 55 (0) | 65 (0) | 650 (0) | 80.19 | 76.26 | 0.05 | 8.21 | 7.80 | 0.05 | | 73.19 | 71.54 | 0.02 | 7.48 | 7.29 | 0.03 | |
| 24 | 2 | 70 (0) | 55 (0) | 10 (−) | 650 (0) | 77.98 | 73.46 | 0.06 | 7.97 | 7.71 | 0.03 | | 73.58 | 71.81 | 0.02 | 7.51 | 7.1 | 0.06 | |
| 25 | 2 | 70 (0) | 100 (+) | 65 (0) | 650 (0) | 86.47 | 84.70 | 0.02 | 16.06 | 15.79 | 0.02 | | 82.54 | 82.84 | 0.00 | 15.33 | 15.05 | 0.02 | |
| 26 | 2 | 70 (0) | 55 (0) | 65 (0) | 650 (0) | 75.27 | 73.05 | 0.03 | 7.73 | 7.91 | 0.02 | | 76.65 | 77.98 | 0.02 | 7.83 | 8.06 | 0.03 | |
| 27 | 2 | 70 (0) | 55 (0) | 65 (0) | 700 (+) | 76.22 | 73.38 | 0.04 | 7.80 | 7.57 | 0.03 | | 79.79 | 79.99 | 0.00 | 8.05 | 7.93 | 0.02 | |
| 28 | 2 | 20 (−) | 55 (0) | 65 (0) | 650 (0) | 58.70 | 54.81 | 0.07 | 18.62 | 17.96 | 0.04 | | 66.78 | 66.72 | 0.00 | 23.87 | 20.66 | 0.16 | |
| 29 | 2 | 120 (+) | 55 (0) | 65 (0) | 650 (0) | 69.26 | 62.99 | 0.10 | 5.25 | 5.08 | 0.03 | | 72.83 | 71.45 | 0.02 | 4.34 | 5.19 | 0.16 | |
| 30 | 2 | 70 (0) | 55 (0) | 65 (0) | 650 (0) | 73.89 | 73.05 | 0.01 | 7.55 | 7.41 | 0.02 | | 79.58 | 77.98 | 0.02 | 8.40 | 8.06 | 0.04 | |

**^1^** Block, 2 Exp: experimental value, ^3^ Prd: predicted value, ^4^ Relative error = $\left| Experimental Value-Predicted Value \right|$

**Table S2.** The absorbance of PRO and PMT individual standard solutions and PRO-PMT mixture at different wavelengths following the adsorption onto BC-OTPR700. Only a sample synthetic mixture is shown.

| Wavelength, λ (nm) | A_PRO – Std_ | A_PMT – Std_ | A_mix_ | A_mix_/A_PRO_ | A_PMT_/A_PRO_ | R^2^ |
| --- | --- | --- | --- | --- | --- | --- |
| 285 | 0.6254 | 0.5941 | 0.0242 | 0.0386 | 0.9500 | 0.9702 |
| 290 | 0.7706 | 0.7134 | 0.0283 | 0.0367 | 0.9258 |  |
| 295 | 0.8894 | 0.7937 | 0.0309 | 0.0348 | 0.8924 |  |
| 300 | 0.9509 | 0.8115 | 0.0313 | 0.0329 | 0.8534 |  |
| 305 | 0.9386 | 0.7625 | 0.0276 | 0.0294 | 0.8124 |  |
| 310 | 0.8662 | 0.6615 | 0.0203 | 0.0235 | 0.7636 |  |

**Table S3.** Confirmatory experiments at the optimum conditions for the removal of PRO and PMT using BC-OTPR700 from single and binary mixtures.

| Trial # | %R_PRO (sin)_ | Mean ±SD | %Er* | %R_PMT (sin)_ | Mean ±SD | %Er* |
| --- | --- | --- | --- | --- | --- | --- |
| 1 | 98.43 | 98.23%±0.38 | 0.42% | 94.97 | 96.03%±0.45 | 0.17% |
| 2 | 98.01 |  |  | 95.68 |  |  |
| 3 | 98.70 |  |  | 95.23 |  |  |
| 4 | 98.19 |  |  | 95.86 |  |  |
| 5 | 97.72 |  |  | 94.83 |  |  |
| Trial # | %R_PRO_ _(bin)_ | Mean ±SD | %Er* | %R_PMT_ _(bin)_ | Mean ±SD | %Er* |
| 1 | 97.17 | 97.43%±0.99 | 1.22% | 94.49 | 95.05%±0.41 | 0.96% |
| 2 | 97.31 |  |  | 95.44 |  |  |
| 3 | 96.08 |  |  | 94.91 |  |  |
| 4 | 98.82 |  |  | 95.50 |  |  |
| 5 | 97.75 |  |  | 95.13 |  |  |
| Trial # | *q_e_* _PRO (sin)_ | Mean ±SD | %Er* | *q_e_* _PMT (sin)_ | Mean ±SD | %Er* |
| 1 | 49.08 | 47.20 mg/g ±1.70 | 0.25% | 38.25 | 38.16 mg/g ±0.64 | 4.22% |
| 2 | 46.91 |  |  | 38.05 |  |  |
| 3 | 47.98 |  |  | 37.29 |  |  |
| 4 | 47.53 |  |  | 38.10 |  |  |
| 5 | 44.51 |  |  | 39.09 |  |  |
| Trial # | *q_e_* _PRO (bin)_ | Mean ±SD | %Er* | *q_e_* _PMT_ _(bin)_ | Mean ±SD | %Er* |
| 1 | 48.58 | 48.71 mg/g ±0.50 | 3.46% | 38.39 | 38.63 mg/g ±0.17 | 3.04% |
| 2 | 48.66 |  |  | 38.77 |  |  |
| 3 | 48.04 |  |  | 38.56 |  |  |
| 4 | 49.41 |  |  | 38.80 |  |  |
| 5 | 48.88 |  |  | 38.65 |  |  |

** Compared to the response value obtained at the optimum conditions, Table 2.*

**Table S4.** BET analysis of BC-OTPR samples.

| Parameters | BC-OTPR600 | BC-OTPR650 | BC-OTPR700 |
| --- | --- | --- | --- |
| Langmuir surface area (m^2^/g) | 21.15 | 35.77 | 53.12 |
| Total pore volume (cm^3^/g) | 0.027 | 0.039 | 0.058 |
| Average pore radius (Å) | 22.5 | 25.1 | 34.5 |
